# Systematic analysis of microorganisms’ metabolism for selective targeting

**DOI:** 10.1038/s41598-024-65936-y

**Published:** 2024-07-16

**Authors:** Mehdi Dehghan Manshadi, Payam Setoodeh, Habil Zare

**Affiliations:** 1 Department of Chemical Engineering, School of Chemical, Petroleum and Gas Engineering, Shiraz University, Shiraz, Iran; 2 W Booth School of Engineering Practice and Technology, McMaster University, Hamilton, ON Canada; 3 Glenn Biggs Institute for Alzheimer’s and Neurodegenerative Diseases, University of Texas Health Science Center, San Antonio, TX USA; 4 Department of Cell Systems and Anatomy, University of Texas Health Science Center, San Antonio, TX USA

**Keywords:** Selective drug target, Synthetic lethality, Narrow-spectrum antibiotic, Organism-specific drug, Genome-scale metabolic model, Systems biology, Computational biology and bioinformatics, Systems biology

## Abstract

Selective drugs with a relatively narrow spectrum can reduce the side effects of treatments compared to broad-spectrum antibiotics by specifically targeting the pathogens responsible for infection. Furthermore, combating an infectious pathogen, especially a drug-resistant microorganism, is more efficient by attacking multiple targets. Here, we combined synthetic lethality with selective drug targeting to identify multi-target and organism-specific potential drug candidates by systematically analyzing the genome-scale metabolic models of six different microorganisms. By considering microorganisms as targeted or conserved in groups ranging from one to six members, we designed 665 individual case studies. For each case, we identified single essential reactions as well as double, triple, and quadruple synthetic lethal reaction sets that are lethal for targeted microorganisms and neutral for conserved ones. As expected, the number of obtained solutions for each case depends on the genomic similarity between the studied microorganisms. Mapping the identified potential drug targets to their corresponding pathways highlighted the importance of key subsystems such as cell envelope biosynthesis, glycerophospholipid metabolism, membrane lipid metabolism, and the nucleotide salvage pathway. To assist in the validation and further investigation of our proposed potential drug targets, we introduced two sets of targets that can theoretically address a substantial portion of the 665 cases. We expect that the obtained solutions provide valuable insights into designing narrow-spectrum drugs that selectively cause system-wide damage only to the target microorganisms.

## Introduction

Elimination of pathogens is the primary goal of any efficient treatment of infection. Broad-spectrum antibiotics have changed the way infectious diseases are treated. They have proven to be a precious tool in the fight against infections, and they are the most promising treatment when the exact identity of the problem-causing pathogen(s) is unknown^1,2^. However, pathogens are not the only targets of broad-spectrum antibiotics. Whether administered orally or not, antibiotics can directly or indirectly influence the human microbiome, which is known as a virtual organ in the human body^2^. The cumulative weight of the human microbiome is comparable to the liver and outnumbers human cells. The human microbiome has been the focus of many studies in recent decades for the etiology of diseases of the liver^3,4^, brain^5–7^, kidney^8,9^, and other human organs^10–12^. These studies suggest the critical role of the microbiome in regulating human health.

The consequences of antibiotic-induced alterations in the microbial composition include variations in microbiota functional characteristics, reduction in microbial diversity, and growth potential for the emergence of drug-resistant microorganisms^13^. For instance, in the short term, fluoroquinolones and β-lactams decreased the diversity of microorganisms by 25% and cut the number of core phylogenetic microbiota from 29 to 12^14^. Additionally, broad-spectrum antibiotics, specifically tetracyclines and macrolides, might be associated with irritable bowel syndrome (IBS) development^15^. Furthermore, the effects of antibiotics on inflammatory bowel disease (IBD)^16,17^, obesity-related disorders^18^, and liver disease^19,20^ have been reported in the literature^21^. On the other hand, narrow-spectrum antibiotics target the specific pathogen that causes the infection. Therefore, many studies suggest using narrow-spectrum antibiotics to overcome the problems related to taking broad-spectrum antibiotics by minimizing the alterations in a patient’s microbiome^1,22–25^.

Identification of selective drug targets facilitates the design of narrow-spectrum antibiotics. However, finding potential drug targets is the first step toward obtaining selective ones. Different methods for identifying potential drug targets use computational approaches^26^. Development of genome-scale metabolic network reconstructions (GENREs) has significantly contributed to a vast branch of computational approaches, such as in silico strain design^27–31^ and drug target identification^32–35^. Using genome-scale metabolic models (GEMMs) as mathematical representatives of GENREs^36–38^ and applying constraint-based computational methods such as flux balance analysis (FBA)^39^, numerous drug targets for *Escherichia coli*, *Helicobacter pylori*, *Mycobacterium tuberculosis, Staphylococcus aureus*^32^, *Acinetobacter baumannii* AYE^40^, and *Pseudomonas aeruginosa* PAO1^41^ have been identified. Among these studies, Lee et al.^32^ provided selective drug targets for four pathogens through the metabolite-centric approach and choke-point analysis^42^. However, none of these studies has investigated the challenges of conserving one or more microorganisms while targeting the others.

**Figure 1:**
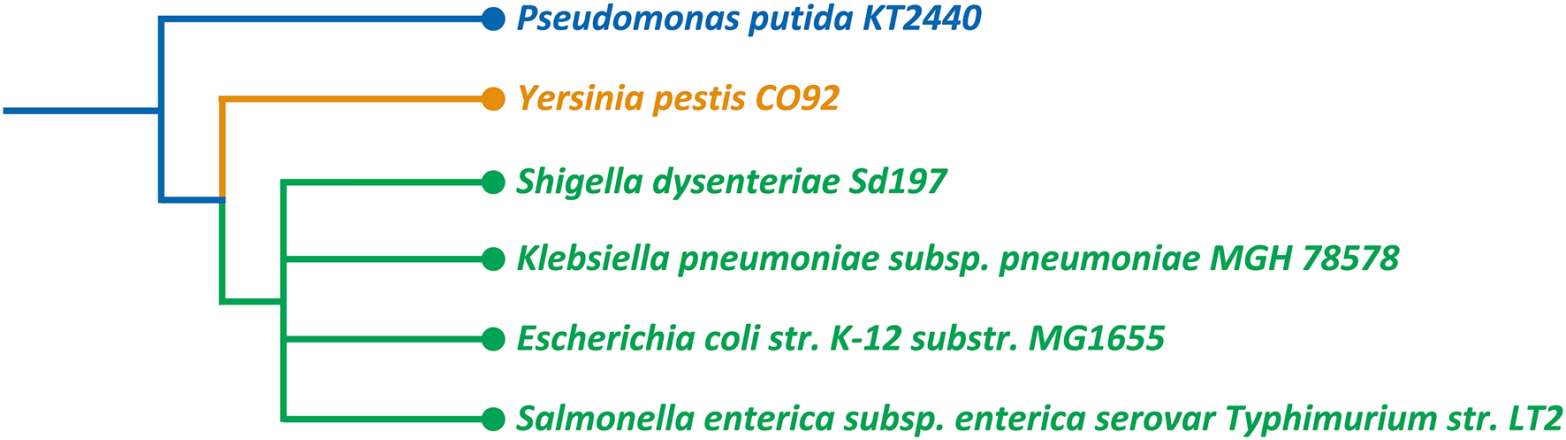
The phylogenetic tree of the six microorganisms generated by iTOL^48^. According to this tree, *P. putida* is the most different strain among these six microorganisms. Furthermore, *S. dysenteriae*, *K. pneumoniae*, *E. coli*, and *S. enterica* stand at the same level.

According to the literature, multi-target drugs are more efficient in competing against infectious pathogens, especially drug-resistant microorganisms^43–45^. The concept of synthetic lethality and identification of synthetic lethal sets pave the way for designing multi-target drugs. Synthetic lethality is a concept where the combination of two non-lethal mutations results in cell death, while each mutation alone is survivable. This concept can be expanded to higher-order synthetic lethality with more than two targets. In this study, we combined the idea of synthetic lethality with selective drug targeting to obtain multi-target, microorganism-specific potential drug candidates for six different microorganisms depicted in Fig. 1. Accordingly, we explored all possible combinations of these six microorganisms, creating scenarios where they are either targeted or preserved in groups ranging from one to six members. By ensuring at least one targeted microorganism in each group, we generated 665 combinations, each referred to as a case. Among these, six cases involve targeting only a single microorganism. For these single-target cases, we identified individual essential reactions as well as double, triple, and quadruple synthetic lethal sets. These solutions were determined in models constrained by rich medium conditions and high oxygen availability, representing their most resilient state. For the remaining 659 cases, we analyzed the solutions identified in the single-target cases to find essential reactions and synthetic lethal sets common to the targeted microorganisms. We ensured these solutions had no detrimental effects on the conserved microorganisms in their most vulnerable state. In other words, the neutrality of solutions for conserving strains was tested under minimal medium conditions with minimum oxygen availability. Additionally, we conducted an analysis to understand the role and contribution of different pathways in the obtained drug targets. We also introduced two sets of reactions capable of handling a considerable portion of all cases.

This study advances selective antimicrobial therapy by identifying numerous potential drug targets and synthetic lethal sets for various microorganisms. By focusing on multi-target sets, we can target subtle metabolic differences between similar microorganisms, which is crucial for developing narrow-spectrum antibiotics. These targeted approaches minimize alterations to the patient's microbiome, reducing the risk of adverse effects from broad-spectrum antibiotics, which negatively affect useful bacteria. The identified targets provide valuable clues for further experimental trials, aiding in the design of narrow-spectrum drugs that selectively cause system-wide damage only to the target microorganisms.

## Methods

We organized our study into four steps and explained each step in detail in this section.

### First step: preparing genome-scale metabolic models

We considered a group of six microorganisms listed in Table 1. We selected these six models based on their size and their considerable number of common reactions. The selected models exhibit a remarkable level of consistency, with a total of 1048 common reactions across all six microorganisms. This shared set of reactions accounts for approximately 35% to 50% of the total reactions present in each individual model. This considerable overlap underscores the fundamental metabolic pathways and core functions that are shared among these microorganisms, despite their distinct genetic makeup and ecological niches.

**Table 1:** List of the studied microorganisms and their GEMMs. The models are provided from the BiGG database^46^.

Microorganism name	Model name	No. of metabolic genes	No. of metabolic reactions	Ref.
*Klebsiella pneumoniae*	iYL1228	1229	2262	^49^
*Pseudomonas putida*	iJN1463	1462	2927	^50^
*Escherichia coli*	iML1515	1516	2712	^51^
*Salmonella enterica*	STM_v1_0	1271	2545	^52^
*Shigella dysenteriae*	iSDY_1059	1059	2539	^53^
*Yersinia pestis*	iPC815	815	1961	^54^

The selection of these six microorganisms was mainly influenced by the lack of extensively curated models for microorganisms. Among these, only *Escherichia coli strain* K-12 substr. MG1655 and *Pseudomonas putida* KT2440 are non-pathogenic^47^. Once comprehensive and reliable curated models for other non-pathogenic microorganisms become available, our approach can be readily applied to include them in similar analyses. Figure 1 shows the phylogenetic tree of these six microorganisms, generated using iTOL^48^.

Based on the availability of nutrition and oxygen, we defined four different media for each microorganism imposing the relevant constraints^55^: (a) a rich medium with a high oxygen uptake rate (R–H), (b) a rich medium with a low oxygen uptake rate (R-L), (c) a minimal medium with high oxygen uptake rate (M–H), and (d) a minimal medium with low oxygen uptake rate (M–L). However, we only focused on the R–H and M–L media as the cells' most resilient and vulnerable states, respectively.

### Second step: identification of synthetic lethal sets

We used the concept of synthetic lethality^56–60^ to provide potential multi-target drugs. We used the Rapid-SL algorithm^61^ to identify these synthetic lethal sets. This method deploys the depth-first-search approach to examine all potential combinations efficiently. To specify and reduce the search space for Rapid-SL, we excluded all non-gene-associated reactions, exchange reactions, demand reactions, spontaneous reactions, and diffusion reactions, which also helped us to reduce the number of not-applicable and trivial solutions. We should first obtain single essentials and synthetic lethal sets for each model. Subsequently, we can analyze these solutions to find selective drug targets in the third step.

An effective potential drug target was defined as a single or synthetic lethal reaction set if it was deleterious for all four distinct medium conditions. However, there was no need to perform a lethality analysis for each medium condition separately to find common solutions. Because the solution space of the R-H medium is a subset of other medium conditions, any lethal set identified for the R-H medium is deleterious in the other medium conditions. Therefore, we performed the lethality analysis of each model just for the R-H medium condition.

### Third step: identification of potential microorganism-specific and multi-target drugs

**Figure 2:**
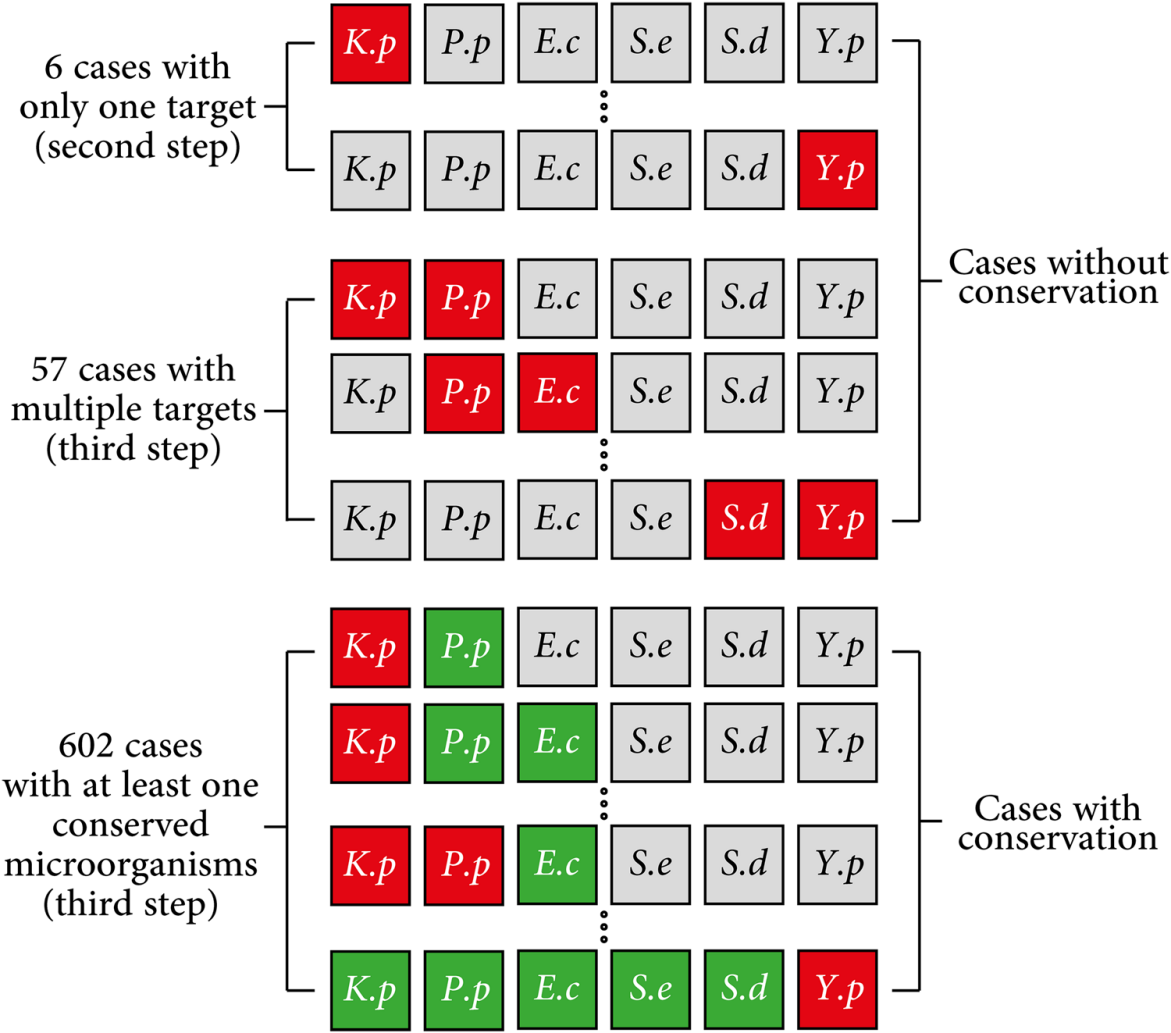
The schematic of the different cases that are considered in this study. *K.p, P.p, E.c, S.e, S.d*, and *Y.p* stand for *Klebsiella pneumoniae, Pseudomonas putida, Escherichia coli, Salmonella enterica, Shigella dysenteriae,* and *Yersinia pestis*, respectively. For each case, the targeted microorganisms are shown in red and the conserved ones are shown in green. The microorganisms that are not included, are shown in gray. This figure categorizes the cases into three groups: six cases with only one targeted microorganism, 57 cases with two to six targeted microorganisms, and 602 cases with at least one conserved microorganism.

Here, we investigated various cases as groups of two to six microorganisms generated by categorizing microorganisms as targeted and conserved. Accordingly, 659 individual combinations were made, and in 57 cases, we examined the targeting of two to six microorganisms while none was conserved. In the other 602 cases, we considered at least one targeted and one conserved microorganism. Figure 2 shows a schematic of the different studied cases.

In this step, we checked the selectivity of each solution obtained in the third step for different case studies. We defined selective solutions as single lethal or synthetic lethal reaction sets that are deleterious for all targeted microorganisms but not for the conserved ones. However, to ensure that these selective solutions are non-lethal for all medium conditions, we tested their knockout effects for the M-L medium condition of all conserved models. This constraint guarantees that conserved microorganisms survive in their most vulnerable state, and the obtained potential solution remains selective.

### Fourth step: applying other strict constraints

There are two possibilities when a selective solution is found: (a) all target reactions are present in all conserved models, or (b) at least one target reaction is not present in one of the conserved models. In both scenarios, the knockout of target reactions is not deadly for the conserved models. However, these models only cover a fraction of their whole genome. Therefore, in the second scenario, it is probable that the target reactions and their corresponding genes are not considered in the model while they are present in the conserved microorganism's genome. Based on this fact, we cautiously pretermitted the selective solutions obtained from the second scenario due to the lack of accurate information.

## Results

We investigated 665 cases covering all combinations of targeted and conserved microorganisms across the six models depicted in Fig. 1. Among the 665 studied cases, 63 focused only on targeting different microorganisms without conserving any, while in the other 602, at least one microorganism was conserved along with other targeted microorganisms. We categorized these two groups of case studies as *with-conservation* and *without-conservation*, respectively.

Here we reported and analyzed the results of the case studies considering the constraints of the fourth step. Both the results of the third and fourth steps are reported in Supplementary files S1, S2, and S3. Note that the two labels of *targeted* and *conserved* were considered for both pathogens and non-pathogens in this work and for specific applications, a group of non-pathogenic microorganisms could be studied to exclusively target non-pathogenic microorganisms. Moreover, our approach is based solely on *in silico* experiments and further experimental validation will be needed to prove the efficacy and safety of all the identified solutions.

### Investigating the results of specific case studies

#### Targeting all but one microorganism

The first row of Table 2 shows the number of potential solutions for simultaneously targeting all six microorganisms. Other rows show the number of identified potential solutions for selectively targeting five out of six microorganisms while conserving the other one. Table 2 reveals that numerous possible solutions are available to kill all six microorganisms simultaneously; however, conserving even one of the microorganisms extremely reduces the number of possible solutions, which agrees with the unanimous idea that selective targeting is challenging.

**Table 2:** The number of potential drug targets identified for targeting five microorganisms while conserving one microorganism. Available potential solutions decreased drastically compared to the number of potential solutions for targeting all six microorganisms (the first row).

Name of the conserved strain	Number of potential solutions				
	Single essentials	Double SLs	Triple SLs	Quadruple SLs	Total solutions
no conservation	31	196	731	1,033	1,991
*Y. pestis*	19	0	1	31	51
*S. dysenteriae*	0	0	7	3	10
*S. enterica*	0	0	0	0	0
*E. coli*	3	12	27	16	58
*P. putida*	9	5	31	16	61
*K. pneumoniae*	19	3	0	17	39

#### Conserving all but one microorganism

Table 3 shows the number of the identified solutions in six cases where all but one microorganism are conserved. This table shows a relatively larger number of potential selective drug targets for *P. putida*, which suggests phylogenetic differences can lead to ease of the selective targeting of this microorganism (Fig. 1)*.*

**Table 3:** The number of potential drug targets identified for conserving five microorganisms while targeting one particular microorganism. Targeting *P. putida* has more potential solutions possibly due to its phylogenetic differences.

Name of the targeted strain	Number of potential solutions				
	Single essentials	Double SLs	Triple SLs	Quadruple SLs	Total solutions
*Y. pestis*	2	4	0	8	14
*S. dysenteriae*	0	0	3	12	15
*S. enterica*	2	0	0	0	2
*E. coli*	1	0	0	0	1
*P. putida*	28	19	25	13	85
*K. pneumoniae*	0	2	5	8	15

#### Cases with no potential solutions

There were 52 cases for which we did not find any potential solutions. In all of these cases, we were aiming to conserve microorganisms that were at the same level of the phylogenetic tree as the targeted microorganisms. All solutions for some of these cases were pretermitted after applying the strict constraints of the fourth step. However, 34 cases remained without any potential solutions, even without these constraints. In all of these cases, at least one microorganism was conserved. Only *S. enterica* was present in all 34 cases, mostly as a *conserved* strain (28 cases). Rapid-SL identified a considerably larger number of essential reactions for *S. enterica* compared to the other microorganisms under study. This issue leads to a considerable overlap between the potential solutions for *S. enterica* and other similar microorganisms (i.e., *K. pneumoniae, E. coli, and S. dysenteriae*). Consequently, gene or gene sets targeting both *K. pneumoniae* and *E. coli,* or *K. pneumoniae* and *S. dysenteriae,* were found to be deadly for *S. enterica*. The detailed results of these cases that had no solution are listed in the Supplementary file S4.

### Mapping the identified solutions to pathways

In this section, we determined which pathways are more suitable for the selective targeting of microorganisms. To this aim, we marked the pathways involved based on the target reactions in each case study. We found 49 different pathways that participated in at least one potential solution. The participation rate of each pathway is reported in Supplementary file S5. In the following, we specifically analyze some of these pathways.

#### The most targeted pathways

Cell envelope biosynthesis, glycerophospholipid metabolism, membrane lipid metabolism, and nucleotide salvage pathway are four pathways participating in all 63 cases of *without-conservation*. These pathways are related to the cell wall/membrane synthesis and the DNA replication of the microorganisms. Manipulating these pathways is the most common mechanism of available antibiotics^62,63^. These pathways are also the most frequently attacked in *with-conservation* cases relative to other pathways. Table 4 shows the number of *with-conservation* cases that can be accomplished by attacking these four pathways showing that no microorganism could be selectively conserved while an essential reaction from the membrane lipid metabolism pathway is targeted. In other words, these microorganisms share the same essential reactions from the membrane lipid metabolism pathway, and consequently, this pathway cannot offer any essential reaction for *with-conservation* cases.

**Table 4:** The list of pathways that appear in all 602 *with-conservation* cases. The numbers show how many cases can be accomplished by attacking the corresponding pathway. For example, 122 of 602 cases can be fulfilled by attacking the essential reactions of the Nucleotide salvage pathway.

Pathway names	Single Essentials	Double SLs	Triple SLs	Quadruple SLs
Cell envelope biosynthesis	215	201	235	288
Glycerophospholipid metabolism	185	189	159	139
Membrane lipid metabolism	0	141	193	276
Nucleotide salvage pathway	122	181	220	190

#### Pathways targeted only by synthetic lethal sets

Among all solutions found, pathways such as cysteine metabolism, fatty acid biosynthesis, pyruvate metabolism, etc. were attacked only by synthetic lethal sets that include two or more reactions (i.e., none of these pathways were targeted by a single lethal reaction). In addition, no synthetic lethal set was found that maps to only one of these pathways. Supplementary file S6 tabulates 15 pathways that have the same characteristics. Note that some synthetic lethal sets map to only one pathway, such as the pentose phosphate or glycolysis and gluconeogenesis pathways.

### Key reactions

It is valuable to find a relatively small group of reactions, the subsets of which could form different individual solutions capable of handling a considerable portion of the studied cases. Of course, obtaining the best solution for this problem is not easy and requires addressing an optimization problem that is out of the scope of this work. However, identifying near-optimal solutions is possible. We applied the greedy algorithm^64^ to find a collection of reactions whose subsets could handle a large portion of the 665 cases. We found many four-membered collections that can cover more than 30% of all cases. As an instance, we reported a particular collection of single lethals that fulfills 198 cases, already available drugs for these reactions, and their corresponding gene-protein-reaction (GPR) relationships (Table 5). The reported drug names are extracted from the DrugBank database^65^.

**Table 5:** A group of four single lethals to fulfill 198 cases. Reaction abbreviations are the reaction IDs in the genome-scale models. For each reaction, only one drug is reported, which targets the associated proteins(s) or genes(s).

Reactions abbreviation	Details	
UAGCVT	GPR	*murA*
	Protein	UDP-N-acetylglucosamine 1-carboxyvinyltransferase
	Pathway	Cell Envelope Biosynthesis
	Known drug	Uridine-Diphosphate-N-Acetylglucosamine
3OAS161	GPR	*fabB*
	Protein	3-oxoacyl-[acyl-carrier-protein] synthase I
	Pathway	Cell Envelope Biosynthesis
	Known drug	Lauric acid
RBFSa	GPR	*ribE*
	Protein	6,7-dimethyl-8-ribityllumazine synthase
	Pathway	Cofactor and Prosthetic Group Biosynthesis
	Known drug	Dithioerythritol
IMPD	GPR	*guaB*
	Protein	IMP dehydrogenase
	Pathway	Purine and Pyrimidine Biosynthesis
	Known drug	Inosinic Acid

Some examples of these 198 cases follow. The deletion of the 3OAS161 reaction or its associated gene,  *fabB*, targets four specific microorganisms: *K. pneumoniae*, *P. putida*, *S. enterica*, and *Y. pestis*, while conserving *E. coli*. Deleting the IMPD reaction or its associated gene, *guaB*, can fulfill several cases including: a) targeting *K. pneumoniae* and *S. dysenteriae*, while conserving *P. putida,* and b) targeting *S. dysenteriae* and *Y. pestis,* while conserving both *P. putida* and *E. coli.* Each target listed in Table 5 is a single lethal reaction. As expected, the application of some of these single essential targets is limited due to drug resistance^66–68^. Therefore, we searched for other collections composed of only synthetic lethal sets based on the hypothesis that attacking microorganisms with multiple targets increases the chance of overcoming drug resistance. We obtained a collection of seven reactions whose subsets can fulfill 236 cases (Table 6).

**Table 6:** Subsets of these seven reactions can fulfill 236 cases.

Reactions abbreviation	Details		
PGI	GPR	*pgi*	
	Protein	Glucose-6-phosphate isomerase	
	Pathway	Glycolysis/Gluconeogenesis	
	Known drug	5-Phosphoarabinonic acid	
PGK	GPR	*pgk*	
	Protein	Phosphoglycerate kinase	
	Pathway	Glycolysis/Gluconeogenesis	
	Known drug	–	
TPI	GPR	*tpiA*	
	Protein	Triose-phosphate isomerase	
	Pathway	Glycolysis/Gluconeogenesis	
	Known drug	–	
FBA	GPR	*fbaA* or *fbaB*	
	Protein	Fructose-bisphosphate aldolase class I & II	
	Pathway	Glycolysis/Gluconeogenesis	
	Known drug	Phosphoglycolohydroxamic Acid	
GND	GPR	*gnd*	
	Protein	6-phosphogluconate dehydrogenase	
	Pathway	Pentose Phosphate Pathway	
	Known drug	–	
TKT2	GPR	*tktA* or *tktB*	
	Protein	Transketolase	
	Pathway	Pentose Phosphate Pathway	
	Known drug	Cocarboxylase	
PGL	GPR	*pgl*	
	Protein	6-phosphogluconolactonase	
	Pathway	Pentose Phosphate Pathway	
	Known drug	Formic acid	

This collection includes e.g., three distinct synthetic lethal sets: (1) a double synthetic lethal set of TKT2 and PGL, which allows us to only target *Y. pestis* while conserving all others, (2) a triple set of PGI, TPI, and GND, which targets only *S. dysenteriae* while conserving all other five microorganisms, and (3) a quadruple set of PGK, TKT2, TPI, and FBA, which is capable of conserving *E. coli* while targeting all other five microorganisms. Consider that other combinations between these seven reactions, such as PGK, TKT2, and TPI can fulfill other cases, such as conserving *E. coli* while targeting *K. pneumoniae*. These seven reactions are highly connected to the central metabolism of microorganisms.

## Discussion

Obtaining selective drug targets is exceptionally challenging compared to identifying non-selective drug targets. In this work, we investigated 665 case studies to introduce selective potential drug targets for combinations of six microorganisms. These cases include 63 *without-conservation* cases, where no microorganism is meant to be conserved, and 602 *with-conservation,* where at least one microorganism in each case is conserved.

According to the results, the cell envelope biosynthesis, glycerophospholipid metabolism, membrane lipid metabolism, and nucleotide salvage pathway contribute in all *without-conservation* cases. Also, these pathways are the targets of many potential solutions for *with-conservation* cases. These pathways are related to cell wall/membrane synthesis and DNA replication, which are reported as the main targets of many antibiotics^62,63^. Furthermore, our findings indicate that while the deletion of up to four reactions within certain pathways, such as cysteine metabolism, fatty acid biosynthesis, or pyruvate metabolism, may not result in a lethal set to halt the growth of the microorganism, the disruption of these pathways in conjunction with other pathways leads to cell death.

It is advantageous to identify a set of targets that can effectively address a significant number of cases. With this objective in mind, we introduced two groups of targets that have the potential to fulfill a substantial portion of the 665 cases under consideration. The first group consists of four essential reactions capable of addressing 198 cases. We also highlighted specific known drugs that target the genes associated with these reactions. As expected, the four essential reactions and their associated genes are susceptible to the development of drug resistance^66–68^. To address this challenge, we identified a second group, including seven reactions composed exclusively of synthetic lethal sets, which can effectively target 236 cases.

One would expect that obtaining synthetic lethal sets provides more potential targets for the cases with no single lethal reactions as solutions. This hypothesis may fit the results of the third step, where several potential synthetic lethal solutions were obtained for 102 cases with no single lethal solution. Also, the results of the third step show that the quadruple synthetic lethal sets generally provide much broader options to accomplish the studied cases. However, applying the strict constraints of the fourth step, which involves considering only the common reactions among the models, eliminates many triple and quadruple synthetic lethal sets. This outcome was predictable because the phylogenetic differences may facilitate the identification of selective drug targets. Consequently, limiting the analysis to common reactions in the fourth step narrows down the acceptable synthetic lethal sets and leaves 54 cases with only single targets as the solution. Thus, the results showed no considerable advantage of synthetic lethal sets over single essentials based on the solutions obtained for the fourth step.

## Conclusion

This study advances selective antimicrobial therapy by identifying numerous potential drug targets and synthetic lethal sets. Targeting subtle metabolic differences between similar microorganisms is key to developing narrow-spectrum antibiotics and minimizing microbiome disruption and adverse effects. These findings offer valuable insights for experimental trials and the design of selective narrow-spectrum drugs. However, due to the lack of highly curated models of non-pathogenic microorganisms in the human gut, we did not split the studied microorganisms into groups of pathogens and non-pathogens. We envision when detailed and accurate models for these microorganisms will become available, our approach would be useful to make microorganism-specific drugs that are not harmful to probiotics and other beneficial gut microorganisms. By conducting targeted studies, we can gain valuable insights into the vulnerabilities unique to particular pathogens, thereby facilitating therapeutic interventions with minimal side effects.

### Supplementary Information

Supplementary Information 1.

Supplementary Information 2.

Supplementary Information 3.

Supplementary Information 4.

Supplementary Information 5.

Supplementary Information 6.

Supplementary Information 7.

## Data Availability

The datasets generated and/or analyzed during the current study are available in the GitHub repository at https://github.com/CSBLaboratory/SelectiveDrugTargets.
