# Supplementary material for: Systematic analysis of microorganisms’ metabolism for selective targeting
Source: Sci Rep. 2024 Jul 16;14:16446. doi: 10.1038/s41598-024-65936-y (PMC11252421; doi:10.1038/s41598-024-65936-y)
Supplement: Supplementary file 1 — Supplementary Information 1. [file 41598_2024_65936_MOESM1_ESM.pdf]

# Systematic analysis of microorganisms' metabolism for selective targeting

Mehdi Dehghan Manshadi, Payam Setoodeh , Habil Zare

## Supplementary Information

**Supplementary file S1:**
An xlsx file. All solutions (combinations of reactions as synthetic lethal sets) found for each case in the third step are listed in this file.

**Supplementary file S2:**
An xlsx file. All solutions (combinations of reactions as synthetic lethal sets) found for each case in the fourth step are listed in this file.

**Supplementary file S3:**
An xlsx file. In this file, the number of solutions for the 665 case studies found in the third and fourth steps are reported.

**Supplementary file S4:**
An xlsx file. In this file, the detailed results of the cases with no solution in the third and fourth steps are reported.

**Supplementary file S5:**
An xlsx file. In this file, the participation rate of each pathway in the obtained solutions are reported.

**Supplementary file S6:**
An xlsx file. In this file, the 15 pathways that are not attacked by any single or synthetic lethal reaction set are reported. In addition, the 18 pathways that can be individually attacked by synthetic lethal sets are also reported.
